# Serum Galectin-3 and Presepsin Levels in Pediatric Familial Mediterranean Fever Patients During Remission: A Prospective Study

**DOI:** 10.3390/diagnostics15182403

**Published:** 2025-09-21

**Authors:** Seyda Dogantan, Peren Perk, Arzu Sekerci Yuksel, Rahime Koc, Adem Keskin

**Affiliations:** 1 Department of Pediatric Rheumatology, Başakşehir Çam ve Sakura City Hospital, Istanbul 34480, Türkiye; rahime-koc@hotmail.com; 2 Institute of Graduate Studies in Health Sciences, Istanbul University, Istanbul 34390, Türkiye; 3 Department of Pediatric Neurology, Başakşehir Çam ve Sakura City Hospital, Istanbul 34480, Türkiye; perkperen@gmail.com; 4 Department of Medical Biochemistry, Başakşehir Çam ve Sakura City Hospital, Istanbul 34480, Türkiye; arzusekerciyuksel@gmail.com; 5 Department of Medical Biochemistry, Faculty of Medicine, Aydin Adnan Menderes University, Aydin 09010, Türkiye; adem.keskin@adu.edu.tr

**Keywords:** familial mediterranean fever, biomarkers, galectin-3, presepsin

## Abstract

**Background/Objectives:** Familial Mediterranean fever (FMF) is the most common hereditary autoinflammatory syndrome, characterized by recurrent fever attacks and serositis. Galectin-3, a β-galactoside-binding lectin involved in inflammation and fibrosis, and presepsin, an established biomarker for bacterial infection and sepsis, have emerged as potential biomarkers for improving diagnostic and prognostic accuracy in autoinflammatory diseases. However, their use in FMF patients is not sufficiently evaluated. This study aims to compare serum galectin-3 and presepsin levels in children with FMF and healthy controls and assess their correlations with conventional acute-phase reactants. **Methods:** This prospective cross-sectional study included 74 children with confirmed FMF during attack-free periods and 67 age- and gender-matched healthy controls. Clinical and genetic characteristics, complete blood count, C-reactive protein (CRP), serum amyloid-A (SAA), and erythrocyte sedimentation rate (ESR) were recorded. Serum galectin-3 and presepsin levels were measured. Group comparisons and correlation analyses were performed using appropriate statistical tests. **Results:** Median serum galectin-3 and presepsin was significantly higher in FMF patients than controls (*p* < 0.001). ESR was significantly higher in FMF patients (*p* < 0.001), while CRP and SAA showed no significant differences. Correlation analysis revealed a strong positive correlation between galectin-3 and presepsin (r = 0.860, *p* < 0.001) in FMF patients, with neither correlating with other acute-phase reactants. **Conclusions:** Galectin-3 and presepsin were found to serve as novel biomarkers reflecting alternative inflammatory pathways in FMF, even during remission. These results, obtained during the attack-free period, indicate the need for further studies to determine the relationship between galectin-3 and presepsin levels and disease activity in FMF.

## 1. Introduction

Familial Mediterranean fever (FMF) is the most common autoinflammatory fever syndrome, characterized by recurrent, spontaneous, and self-limiting fever attacks accompanied by abdominal pain and serositis [1,2]. An increased acute-phase response is typically observed during active disease periods, returning to normal levels during remission periods [3]. However, impaired endothelial function may contribute to persistent low-grade inflammation, as indicated by continuously elevated levels of inflammatory markers [3]. Consequently, distinguishing FMF attacks from infectious episodes or other inflammatory conditions can be challenging when relying solely on conventional acute-phase reactants. In this regard, reliable biomarkers could not only improve the accuracy of FMF diagnosis but also offer prognostic value and improve disease monitoring by providing insights into the underlying inflammatory pathways.Galectin-3, a member of the β-galactoside-binding lectin family, plays a multifaceted role in inflammation, apoptosis, fibrosis, immunity, and tumorigenesis [1,2,4]. Elevated circulating galectin-3 levels have been reported in patients with autoimmune diseases such as systemic lupus erythematosus, rheumatoid arthritis, and FMF, particularly in patients with renal amyloidosis and proteinuria [2,5,6,7]. Galectin-3 has been identified as a novel mediator of kidney fibrosis during chronic inflammatory responses and the progression of chronic kidney disease in FMF patients [2].

Presepsin is a well-established inflammatory biomarker, primarily used for the early diagnosis of sepsis and neutropenia [8,9,10]. Although baseline presepsin levels in healthy individuals are highly variable [8,11], and debate continues on the cut-off value that can be used to differentiate bacterial from non-bacterial systemic inflammation [12], experimental data suggest that elevated presepsin levels may serve as an early marker of infection [13,14]. To our knowledge, no study has evaluated these two biomarkers simultaneously in FMF patients so far. In view of the foregoing, we conducted this study to determine the potential efficacies of galectin-3 and presepsin in the diagnosis and follow-up of FMF by comparing their serum levels between FMF patients and healthy control subjects based on the hypothesis that elevated galectin-3 and presepsin levels could serve as diagnostic and prognostic biomarkers for FMF.

## 2. Materials and Methods

### 2.1. Study Design

This study was designed as an observational, prospective, cross-sectional, single-center study. The study protocol was approved by the Ethics Committee of Başakşehir Çam and Sakura City Hospital (protocol code KAEK-11/28.08.2024.130 and date of approval 2 September 2024). The study was conducted at the Pediatric Rheumatology Department of Başakşehir Çam and Sakura City Hospital between 1 October 2024, and 1 October 2025, per the ethical considerations outlined in the Declaration of Helsinki. Written informed consent was obtained from the legal guardians of the patients prior to the conduct of the study.

### 2.2. Population and Sample

The study population consisted of children aged 2–18 years whose FMF diagnoses were confirmed according to the Eurofever/Pediatric Rheumatology International Trials Organization (PRINTO) criteria [15]. Additionally, the diagnosis of FMF was based on a combination of Tel-Hashomer clinical criteria and genetic analysis [16]. All patients were taking colchicine and other anti-inflammatory or anticoagulant drugs [3,17]. Children younger than 2 years or older than 18 years, those with amyloidosis or proteinuria, those with any chronic comorbid disease, particularly concurrent hematological disorders, those receiving corticosteroid therapy, and those with active infections were excluded from the study [3]. Additionally, single heterozygous patients carrying a variant of uncertain significance (VUS) in the *MEFV* gene were excluded from the study; only those who were homozygous or compound heterozygous were included in the analysis. In the end, the study sample consisted of 74 children in the attack-free period with confirmed FMF diagnosis. On the other hand, the control group consisted of 67 randomly selected healthy children who were consecutively admitted to the General Pediatrics Unit of the same hospital and who did not have any chronic disease, malignancy, hematological or inflammatory condition, or medication use, and who were matched to the patient group for age and gender. Both patient and control groups were divided into three age groups each: early childhood (2–5 years), middle childhood (6–12 years), and adolescence (13–18 years) groups [18].

### 2.3. Data Collection

Demographic characteristics of the children, including BMI (body mass index) sex, and age; clinical characteristics, including exacerbation symptoms and disease duration; laboratory characteristics, including ESR (erythrocyte sedimentation rate), CBC (complete blood count), galectin-3, presepsin, SAA (serum amyloid A), CRP (C-reactive protein), creatinine, BUN (blood urea nitrogen), ALT (alanine aminotransferase), AST (aspartate aminotransferase), and LDH (lactate dehydrogenase) levels; and genetic characteristics, including *MEFV* gene mutations, were prospectively recorded.

Participants’ BMI values were obtained by dividing their body weight in kilograms by the square of their height in meters. Participants’ laboratory tests were determined in the Biochemistry Department of Basaksehir Cam and Sakura City Hospital using their blood samples, which were collected during routine follow-up examinations conducted in the outpatient clinics of the Department. Serum specimens were stored at −80 °C until they were analyzed for SAA and the study-specific biomarkers, namely galectin-3 and presepsin. SAA measurements were performed according to the standard nephelometric method specified in the kit user manual provided by the manufacturer.

Serum values of galectin-3 were analyzed via a commercial ELISA (enzyme-linked immunosorbent assay) kit (SUNRED BIO. 201-12-1952 Human Galectin-3 ELISA Kit, 96T, Assay range: 0.2 ng/mL–60 ng/mL, (Sunred Biological Technology, Shanghai, China)), whereas presepsin levels were measured via an automated immunoassay platform operating with chemiluminescent enzyme-based methodology (SUNRED BIO. 201-12-7968 Human Presepsin ELISA Kit, 96T, Assay range: 40 ng/L–10,000 ng/L, (Sunred Biological Technology, Shanghai, China)). Calibration of the assay was determined per the manufacturer’s recommendations, and galectin-3 concentrations were normalized to a standard curve.

Patients’ familial histories of FMF were obtained from their medical records. For the analysis of *MEFV* gene mutations, genomic DNA was isolated from peripheral blood samples using a commercial isolation kit (QIAamp DNA Blood Mini Kit, QIAGEN GmbH, Hilden, Germany). The *MEFV* gene was analyzed by PCR amplification of the exons harboring common variants, followed by direct Sanger sequencing. Among the 12 commonly targeted variants of *MEFV* (including *M694V*, *M680I*, *M694I*, *V726A*, *A744S*, *R761H*, *E148Q*, *R408Q*, *K695R*, and *P369S*), only ten were detected in our patient cohort (*M694V*, *M680I*, *M694I*, *V726A*, *A744S*, *R761H*, *E148Q*, *R408Q*, *K695R*, and *P369S*). The other targeted variants were not observed in this study population and therefore not listed in the results. The analytical sensitivity of the PCR/Sanger sequencing method for these variants is >99% according to the manufacturer’s specifications and published validation data. Classification and clinical interpretation of the variants were performed in accordance with the ACMG (American College of Medical Genetics and Genomics) guidelines and supported by relevant literature [19,20]. Detected variants were categorized as pathogenic or variants of uncertain significance (VUS).

### 2.4. Study’s Outcomes

The study’s primary outcomes were serum galectin-3 and presepsin levels in children with acute FMF during remission and healthy control subjects, with a view to evaluating their potential prognostic value for predicting disease activity.

The study’s secondary outcomes were the correlations between these potential biomarkers and conventional acute-phase reactants, such as CRP, ESR, and SAA, with a view to exploring their prognostic value in monitoring FMF activity.

### 2.5. Sample Size Calculations

Sample size calculations were performed by comparing the two independent groups using G*Power 3.1.9.2 software. Based on previous studies examining the use of inflammatory biomarkers for predicting autoinflammatory diseases, we anticipated a moderate to large effect size (Cohen’s d = 0.7) to achieve the study’s primary outcomes, namely galectin-3 and presepsin levels. Accordingly, based on a two-tailed significance level of α = 0.05 and a statistical power of 80%, we calculated that at least 34 participants should be included in each study group. Considering a potential 15% dropout rate and participants whose data may not be complete, we aimed to include 80 participants in total, with a minimum of 40 participants in each group.

### 2.6. Statistical Analysis

Statistical analyses of the collected data were performed using Jamovi (version 2.6.44, 2025; available at https://www.jamovi.org, accessed on 15 July 2025) and JASP (version 0.19.3, 2025; available at https://jasp-stats.org, accessed on 15 July 2025) software packages.

The outcomes of the statistical analyses were reported using descriptive measures: for quantitative data following a normal distribution, mean ± standard deviation values were presented; for those not adhering to normality, median values with minimum and maximum ranges were provided; and for categorical data, frequencies (*n*) and percentages (%) were used. The normality of quantitative data was assessed with the Kolmogorov–Smirnov or Anderson–Darling tests, selected according to sample size. The normality assumptions for galectin-3, presepsin, SAA, and other biochemical parameters were also visually examined using histograms and Q-Q plots.

When evaluating differences in categorical data across groups, Pearson’s chi-square test was applied when the expected cell count was five or greater, Fisher’s exact test was employed for 2 × 2 contingency tables with fewer than five expected counts, and the Fisher–Freeman–Halton test was utilized for R × C contingency tables involving multiple categories.

Additionally, when assessing variations in quantitative variables between two unrelated groups, the independent-samples *t*-test was applied to those variables found to follow a normal distribution, whereas the Mann–Whitney U test was employed for data that did not exhibit a normal distribution.

The correlations between inflammatory biomarkers, i.e., galectin-3, presepsin, ESR, CRP, and SAA, in the patient group were evaluated using Spearman correlation analysis for non-normally distributed variables and Pearson’s correlation analysis for normally distributed variables. Statistical significance was determined as a *p*-value ≤ 0.05.

## 3. Results

The distribution of participants’ demographic characteristics by the study groups is given in Table 1. There was no statistically significant difference between the groups in terms of age at admission (*p* = 0.344). More than half of the participants in both groups were in the middle-childhood age group, and there was no significant difference between the groups in terms of age distribution (*p* = 0.892).

The rate of females was 51.4% in the patient group and 58.2% in the control group; there was no significant difference between the groups in terms of gender distribution (*p* = 0.517). There was also no significant difference between the groups in BMI (*p* = 0.084).

**Figure 1:**
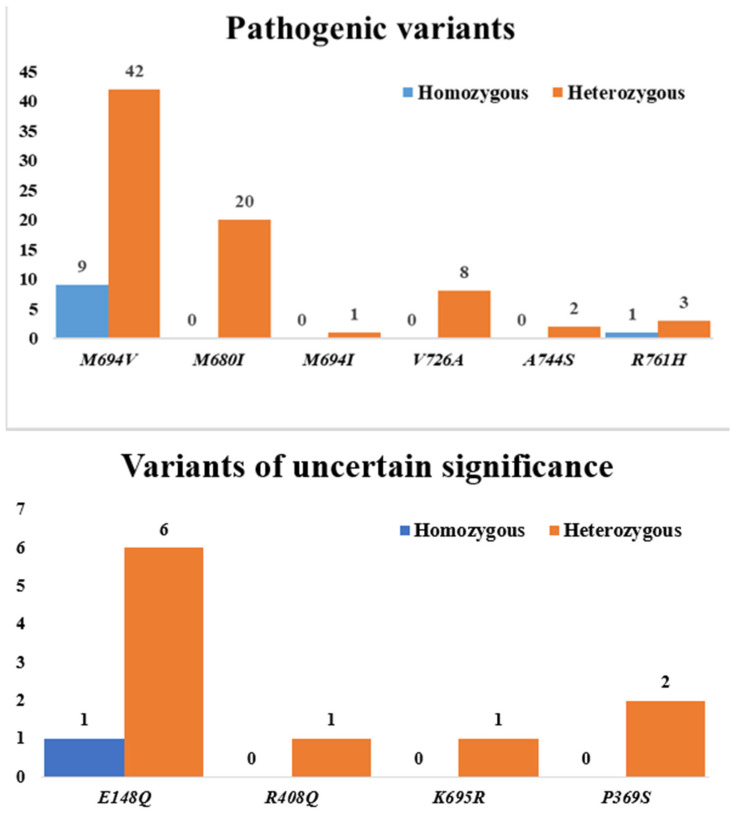
Distribution of *MEFV* gene mutations in the FMF group: pathogenic variants and variants of uncertain significance (VUS).

Patients’ clinical and genetic characteristics are presented in Table 2 and Figure 1. The most frequent *MEFV* gene mutation was *M694V* (68.9%), followed by *M680I* (27.0%). Among FMF-related symptoms, abdominal pain was the most common (78.4%), followed by fever (74.3%) and arthralgia (56.8%).

There was no significant difference between the groups in CBC or other laboratory parameters (*p* > 0.05 for all cases). In terms of renal parameters, the median serum creatinine level of the patient group was significantly higher than that of the control group (*p* < 0.001), while there was no significant difference between the groups in BUN level (*p* = 0.095) (Table 3). As for inflammatory markers, median ESR was significantly elevated in the patient group compared to the control group (8.0 mm/h vs. 3.0 mm/h, *p* < 0.001), while there was no significant difference between the groups in CRP level (*p* = 0.818).

**Figure 2:**
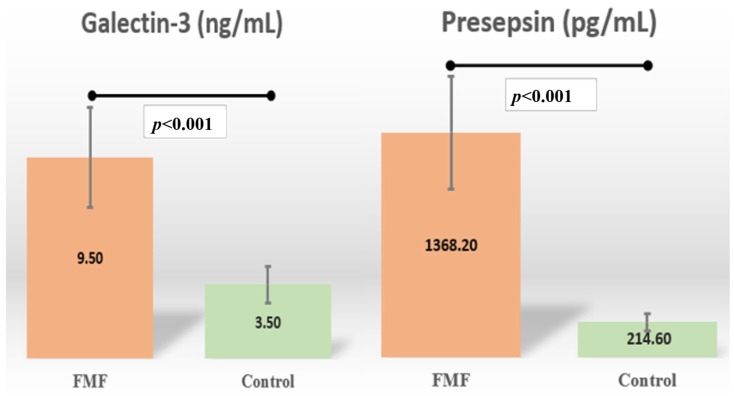
Schematic representation of serum galectin-3 and presepsin levels in the groups. FMF: Familial Mediterranean Fever. The end points of the error bars are determined according to the 25th percentile first quartile (Q1) and 75th percentile third quartile (Q3) values.

The median serum galectin-3 and presepsin level of the patient group was significantly higher than that of the control group (*p* < 0.001) (Figure 2) (Table 3). There was no significant difference between the groups in SAA level (*p* = 0.350) (Table 3).

**Figure 3:**
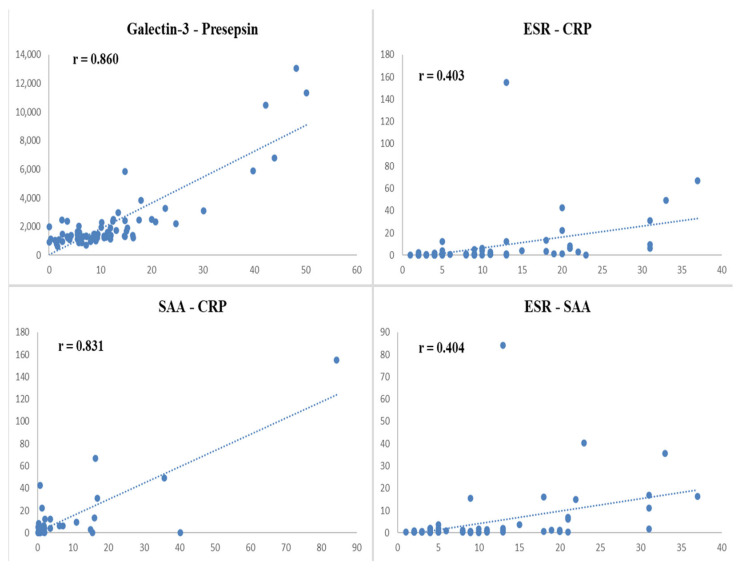
Graphs of Spearman correlation analysis results of some laboratory findings in the Familial Mediterranean Fever group. ESR: Erythrocyte sedimentation rate; SAA: Serum amyloid A; CRP: C-reactive protein; r: Correlation coefficient.

Correlation analysis revealed a strong positive correlation between galectin-3 and presepsin levels (r = 0.860, *p* < 0.001) in FMF patients (Table 4 and Figure 3). ESR was moderately correlated with both SAA (r = 0.404, *p* < 0.001) and CRP (r = 0.403, *p* < 0.001). SAA was strongly correlated with CRP (r = 0.831, *p* < 0.001) (Table 4 and Figure 3). Neither galectin-3 nor presepsin was significantly correlated with conventional acute-phase reactants (*p* > 0.05 for all cases).

When examining the distribution of *MEFV* gene mutations in the patient group, homozygous mutations were detected in 11 patients (14.86%), heterozygous mutations in 42 patients (56.76%), and compound heterozygous mutations in 21 patients (28.38%). The patient group was divided into three subgroups based on the mutation status of the *MEFV* gene: homozygous, heterozygous, and compound heterozygous. FMF family history rates and parameters found to be significant between the patient and control groups were compared at the subgroup level, and the results are presented in Table 5.

No significant difference was found between subgroups in terms of presepsin, galectin-3, and creatinine levels and FMF family history rates (Table 5). A significant difference was found between subgroups in terms of ESR levels (Table 5). ESR levels were higher in the combined heterozygous subgroup than in the heterozygous subgroup (*p* = 0.012).

## 4. Discussion

Serum galectin-3 and presepsin levels were significantly higher in FMF patients during remission than in healthy control subjects, indicating the potential of presepsin and galectin-3 as new biomarkers for predicting disease activity in FMF patients. The fact that these tests yielded significant results despite being performedF during attack-free periods is a remarkable finding in terms of demonstrating the relationship between these biomarkers and disease activity in FMF. Correlation analysis further revealed a strong positive correlation between galectin-3 and presepsin levels in FMF patients, while conventional acute-phase reactants, such as ESR, CRP, and SAA, exhibited interrelated but independent patterns, suggesting that galectin-3 and presepsin may be involved in distinct inflammatory pathways that are not fully captured by traditional markers.

Novel biomarkers, including α9β1 integrin, lymphocyte subgroups, S100A8/A9 and S100A12 proteins, immature granulocytes, circulating endothelial microparticles, and asprosin have been investigated to predict FMF diagnosis and prognosis [3,16,21,22,23,24]. Although promising results have been achieved with these biomarkers, large-scale studies are still needed to validate their diagnostic and prognostic utilities. A few studies have investigated the relationship between galectin-3 and FMF. In one of these studies, Yilmaz et al. [2] reported significantly higher serum galectin-3 levels in FMF patients compared to healthy control subjects, in line with our results. They also found a positive correlation between galectin-3 and proteinuria. We could not examine the relationship between galectin-3 and proteinuria since we excluded children with proteinuria secondary to FMF from our cohort. Batu et al. [1] investigated the efficacy of galectin-3 in differentiating periodic fevers with aphthous stomatitis, pharyngitis, and adenitis (PFAPA) syndrome from FMF. They found that serum galectin-3 did not differ significantly between FMF patients (during attacks or attack-free periods) and healthy control subjects. In contrast, we found significantly elevated galectin-3 levels in FMF patients compared to healthy control subjects, suggesting that disease-specific factors or differences in study design may account for such discrepancies between studies. The small sample size, which might have contributed to the lack of statistical significance, was noted as a limitation in Batu et al.’s study [1].

Galectin-3 has been implicated in various inflammatory and infectious conditions [1,6,25,26,27]. Ten Oever et al. [26] reported higher circulating galectin-3 levels in patients with infections compared to healthy individuals or those with non-infectious inflammatory diseases. However, distinguishing between infectious and autoinflammatory processes based on galectin-3 levels remains a challenge. Prospective studies adjusted for confounding factors, such as patient heterogeneity and disease activity, are needed to clarify the interplay involving galectin-3. Chen and colleagues investigated the relationship between galectin-3 levels and disease activity in Still’s disease, an autoinflammatory disease, and reported that galectin-3 levels were elevated in these patients and positively correlated with IL-1β and IL-18 levels. Furthermore, after 6 months of treatment, galectin-3 levels, clinical activity scores, and plasma IL-1β and IL-18 levels were significantly reduced [5]. In our study, galectin-3 levels were found to be elevated compared to healthy children, despite the patients being in remission. However, they did not show a significant correlation with parameters such as ESR, CRP, or SAA. This may be due to the study being conducted only during the remission period. Furthermore, galectin-3 levels, although measured in remission, showed a high correlation with presepsin levels. Furthermore, we believe that the high positive correlation with presepsin levels, despite galectin-3 levels being measured in remission, merits further investigation to further elucidate the pathophysiological mechanisms and understand its clinical implications.

Presepsin has primarily been studied as a biomarker of infection and sepsis; however, its utility in autoinflammatory disorders, such as FMF, remains poorly defined. While Hashimoto et al. [8] reported that presepsin was ineffective in detecting periprosthetic joint infection without systemic signs, other studies have suggested its prognostic role in febrile neutropenia, lymphoma, and coronavirus disease 2019 (COVID-19) [9,10,28]. Interestingly, a study reported significantly lower presepsin levels in acute cholecystitis patients than in healthy control subjects [14]. On the other hand, in our study, presepsin levels were significantly higher in FMF patients compared to healthy children. This increase was observed during the remission period, suggesting that presepsin is not only a marker associated with acute exacerbations but may also be affected by underlying chronic inflammatory processes.

Our correlation analysis revealed a strong positive relationship between galectin-3 and presepsin in FMF patients, which has not been reported in the literature to date. In contrast, galectin-3 and presepsin did not correlate with traditional acute-phase reactants, a finding consistent with Yılmaz et al. [2]. On the other hand, ESR showed a positive correlation with CRP and SAA, as expected given their known roles as inflammatory markers in FMF. These results suggest that galectin-3 and presepsin may serve as complementary markers to conventional biomarkers in disease monitoring and management.

In our study, subgroups with homozygous, heterozygous, and compound heterozygous mutations were created based on the type of *MEFV* gene mutation, and presepsin and galectin-3 levels were compared in these subgroups. No significant difference was found between the groups. However, ESR levels were higher in children with compound heterozygous mutations than in children with heterozygous mutations. Furthermore, no difference was observed between the rates of family history of FMF in these three subgroups. Finally, children with a single heterozygous VUS (variant of uncertain significance) were excluded from the study.

This study had several limitations. First, the cross-sectional design and the fact that only attack-free FMF patients were included in the sample limited the ability to establish causal relationships or to assess dynamic changes in biomarker levels during disease flares. Future prospective controlled studies, including FMF patients with and without attacks, are warranted to elucidate the pathophysiological roles of these biomarkers. Secondly, the relatively small sample size may have reduced the statistical power to detect subtle differences, particularly for presepsin. Thirdly, not having taken other potential confounding factors, such as subclinical infections or genetic heterogeneity, into account may have affected biomarker levels. Fourthly, the absence of longitudinal follow-up precluded assessing the predictive value of galectin-3 or presepsin for clinical outcomes, including disease severity or amyloidosis risk, to some extent. Finally, the study’s findings were not validated by functional assays or external cohorts, which would have otherwise strengthened their generalizability.

Although our study included only FMF patients during the attack-free period, longitudinal monitoring of galectin-3 and presepsin levels during both attack and remission will further demonstrate the clinical value of these biomarkers. Furthermore, the fact that galectin-3 and presepsin levels have been investigated in other autoinflammatory syndromes such as PFAPA and Still’s disease suggests that these biomarkers may be elevated in different diseases. Therefore, comparative studies are needed to clearly determine specificity. If clinically validated in larger, multicenter patient groups, galectin-3 and presepsin could be valuable tools in addition to traditional markers (CRP, SAA) for the detection of subclinical inflammation, early diagnosis, and monitoring of treatment response.

## 5. Conclusions

In conclusion, despite these limitations, our findings suggest that galectin-3 and presepsin may serve as a potential biomarker for FMF. The high correlation between galectin-3 and presepsin, and the lack of correlation between these parameters and conventional acute-phase reactants, suggest that presepsin and galectin-3 may reflect alternative inflammatory pathways. Compared with the sensitive but nonspecific inflammatory markers CRP and SAA, presepsin and galectin-3 may offer additional insights into the fibrotic and immune-mediated processes underlying FMF pathogenesis. Their high levels in our cohort, even during attack-free periods, suggest that presepsin and galectin-3 may predict subclinical inflammation and potentially aid in early diagnosis or risk stratification. Future multicenter, longitudinal studies are needed to assess whether galectin-3 and presepsin can complement established markers such as CRP and SAA in guiding treatment decisions and monitoring disease activity.

## Figures and Tables

**Table 1:** Comparison of demographic characteristics between children with Familial Mediterranean Fever and healthy controls.

		FMF Group (*n* = 74)	Control Group (*n* = 67)	*p* *
Age at admission (year) X ± SD		10.8 ± 4.1	10.1 ± 4.1	0.344
Age groups *n* (%)	Early childhood (2–5 years)	10 (13.5)	7 (10.4)	0.892
	Middle childhood (6–12 years)	37 (50.0)	37 (55.2)	
	Adolescents (13–18 years)	27 (36.5)	23 (34.3)	
Sex *n* (%)	Female	38 (51.4)	39 (58.2)	0.517
	Male	36 (48.6)	28 (41.8)	
Body mass index (kg/m^2^) X ± SD		18.5 ± 3.8	17.5 ± 3.3	0.084

FMF: Familial Mediterranean Fever, X ± SD: Mean ± Standard Deviation. *: Independent Samples *t*-Test was used to compare continuous variables, and Pearson’s Chi-Square test was used to compare categorical variables.

**Table 2:** *MEFV* gene mutation spectrum and clinical manifestations in children with Familial Mediterranean Fever.

Familial Mediterranean Fever Group (*n* = 74)					
*MEFV* Gene Mutation Spectrum					
Variants	*MEFV* Mutations		*n* (%) ^Ω^	Mutation Types	
Pathogenic variants	*M694V*		51 (68.9)	Homozygous *n* (%)	9 (17.6)
				Heterozygous *n* (%)	42 (82.4)
	*M680I*		20 (27.0)	Homozygous *n* (%)	0
				Heterozygous *n* (%)	20 (100)
	*M694I*		1 (1.4)	Homozygous *n* (%)	0
				Heterozygous *n* (%)	1 (100)
	*V726A*		8 (10.8)	Homozygous *n* (%)	0
				Heterozygous *n* (%)	8 (100)
	*A744S*		2 (2.7)	Homozygous *n* (%)	0
				Heterozygous *n* (%)	2 (100)
	*R761H*		4 (5.4)	Homozygous *n* (%)	1 (25)
				Heterozygous *n* (%)	3 (75)
Variants of uncertain significance	*E148Q*		7 (9.5)	Homozygous *n* (%)	1 (14.3)
				Heterozygous *n* (%)	6 (85.7)
	*R408Q*		1 (1.4)	Homozygous *n* (%)	0
				Heterozygous *n* (%)	1 (100)
	*K695R*		1 (1.4)	Homozygous *n* (%)	0
				Heterozygous *n* (%)	1 (100)
	*P369S*		2 (2.7)	Homozygous *n* (%)	0
				Heterozygous *n* (%)	2 (100)
Clinical manifestations					
Family history of FMF *n* (%)				55 (74.3)	
Main presenting symptom *n* (%) ^Ω^		Abdominal pain		58 (78.4)	
		Arthralgia		42 (56.8)	
		Chest pain		25 (33.8)	
		Fever		55 (74.3)	
		Headache		9 (12.2)	
		Nausea/vomiting		9 (12.2)	
		Diarrhea		3 (4.1)	

FMF: Familial Mediterranean Fever; *MEFV*: Mediterranean Fever gene. ^Ω^: Because some patients had compound heterozygous mutations and/or multiple symptoms, these patients were included more than once in the percentile frequency of different mutation types and symptoms.

**Table 3:** Laboratory findings of the groups.

	FMF Group (*n* = 74)	Control Group (*n* = 67)	*p* *
Hemoglobin (g/dL) ^§^	12.4 [5.2–16.0]	12.6 [5.2–15.2]	0.843
Leukocyte count (10^3^/μL) ^§^	7.2 [3.9–12.6]	7.2 [5.2–11.4]	0.806
Platelet count (10^3^/μL) ^§^	325.0 [192.0–627.0]	330.0 [192.0–627.0]	0.934
Blood urea nitrogen (mg/dL) ^§^	22.7 [13.3–39.8]	23.5 [15.1–39.8]	0.095
Creatinine (mg/dL) ^§^	0.5 [0.3–0.9]	0.3 [0.1–0.7]	<0.001
Alanine aminotransferase (U/L) ^§^	15.0 [6.0–40.0]	14.0 [7.0–40.0]	0.432
Aspartate aminotransferase (U/L) ^§^	22.0 [12.0–43.0]	20.0 [16.0–43.0]	0.285
Lactate dehydrogenase (U/L) ^§^	210.0 [138.0–347.0]	203.0 [150.0–324.0]	0.733
Erythrocyte sedimentation rate (mm/h) ^§^	8.0 [1.0–37.0]	3.0 [1.0–9.0]	<0.001
C-reactive protein (mg/L) ^§^	0.6 [0.1–155.0]	1.0 [0.2–6.5]	0.818
Serum amyloid A (mcg/mL) ^§^	0.5 [0.2–84.3]	0.4 [0.2–3.5]	0.350
Galectin-3 (ng/mL) ^§^	9.3 [0.0–50.2]	3.5 [1.2–29.7]	<0.001
Presepsin (pg/mL) ^§^	1368.2 [610.7–13,066.0]	214.6 [24.2–2022.8]	<0.001

FMF: Familial Mediterranean Fever, ^§^: Median [Minimum–Maximum]. *: Mann–Whitney U test.

**Table 4:** Spearman correlation analysis results of some laboratory findings in the Familial Mediterranean Fever group.

	Presepsin	ESR	SAA	CRP
Galectin-3	r = 0.860	r = 0.088	r = −0.019	r = −0.030
	*p* < 0.001	*p* = 0.457	*p* = 0.871	*p* = 0.801
Presepsin	-	r = 0.035	r = 0.021	r = −0.001
		*p* = 0.766	*p* = 0.862	*p* = 0.992
ESR	-	-	r = 0.404	r = 0.403
			*p* < 0.001	*p* < 0.001
SAA	-	-	-	r = 0.831
				*p* < 0.001

ESR: Erythrocyte sedimentation rate; SAA: Serum amyloid A; CRP: C-reactive protein; r: Correlation coefficient.

**Table 5:** Analysis of laboratory parameters in subgroups of the patient group depending on the *MEFV* gene mutation type.

	Homozygous Subgroup (*n* = 11)	Heterozygous Subgroup (*n* = 42)	Compound Heterozygous Subgroup (*n* = 21)	*p* *
Family history of FMF *n* (%)	8 (72.7)	33 (78.6)	14 (66.7)	0.589
Creatinine (mg/dL) ^§^	0.6 [0.3–0.8]	0.5 [0.3–0.9]	0.5 [0.3–0.8]	0.297
Erythrocyte sedimentation rate (mm/h) ^§^	5.0 [2.0–31.0]	5.0 [1.0–37.0]	11.0 [4.0–31.0]	0.042
Galectin-3 (ng/mL) ^§^	7.3 [1.2–50.2]	9.4 [0.3–43.9]	10.8 [0.04–22.7]	0.903
Presepsin (pg/mL) ^§^	1372.3 [718.6–13,066.0]	1395.0 [767.4–110,497.4]	1360.0 [610.7–3255.7]	0.775

FMF: Familial Mediterranean Fever, ^§^: Median [Minimum–Maximum], *. Kruskal–Wallis test was used to compare continuous variables, and Pearson’s Chi-Square test was used to compare categorical variables.

## Data Availability

The datasets generated and analyzed during the current study are not publicly available due to patient privacy regulations but are available from the corresponding author upon reasonable request and with appropriate ethical approvals.

## Abbreviations

The following abbreviations are used in this manuscript:

ALT	Alanine aminotransferase
AST	Aspartate aminotransferase
BMI	Body mass index
BUN	Blood urea nitrogen
CBC	Complete blood count
COVID-19	Coronavirus disease 2019
CRP	C-reactive protein
ELISA	Enzyme-linked immunosorbent assay
ESR	Erythrocyte sedimentation rate
FMF	Familial Mediterranean fever
LDH	Lactate dehydrogenase
*MEFV*	Mediterranean fever gene
PFAPA	Periodic fever, aphthous stomatitis, pharyngitis, and adenitis
PRINTO	Pediatric Rheumatology International Trials Organization
SAA	Serum amyloid A
